# Rapid molecular evolution of *Spiroplasma* symbionts of *Drosophila*

**DOI:** 10.1099/mgen.0.000503

**Published:** 2021-02-16

**Authors:** Michael Gerth, Humberto Martinez-Montoya, Paulino Ramirez, Florent Masson, Joanne S. Griffin, Rodolfo Aramayo, Stefanos Siozios, Bruno Lemaitre, Mariana Mateos, Gregory D. D. Hurst

**Affiliations:** ^1^​ Institute of Infection, Veterinary and Ecological Sciences, University of Liverpool, Liverpool, UK; ^2^​ Laboratorio de Genética y Genómica Comparativa, Unidad Académica Multidisciplinaria Reynosa Aztlán, Universidad Autónoma de Tamaulipas, Reynosa, Mexico; ^3^​ Department of Cell Systems and Anatomy, University of Texas Health San Antonio, San Antonio, TX, USA; ^4^​ Global Health Institute, School of Life Sciences, Swiss Federal Institute of Technology Lausanne (École Polytechnique Fédérale de Lausanne), Lausanne, Switzerland; ^5^​ Department of Biology, Texas A&M University, College Station, TX, USA; ^6^​ Department of Ecology and Conservation Biology, Texas A&M University, College Station, TX, USA; ^†^​ Present address: Department of Biological and Medical Sciences, Oxford Brookes University, Oxford, UK

**Keywords:** DNA repair, genome evolution, genome reduction, mycoplasma, symbiosis

## Abstract

*
Spiroplasma* is a genus of *Mollicutes* whose members include plant pathogens, insect pathogens and endosymbionts of animals. *Spiroplasma* phenotypes have been repeatedly observed to be spontaneously lost in *Drosophila* cultures, and several studies have documented a high genomic turnover in *Spiroplasma* symbionts and plant pathogens. These observations suggest that *Spiroplasma* evolves quickly in comparison to other insect symbionts. Here, we systematically assess evolutionary rates and patterns of *Spiroplasma poulsonii *, a natural symbiont of *Drosophila*. We analysed genomic evolution of *s*Hy within flies, and *s*Mel within *in vitro* culture over several years. We observed that *S. poulsonii* substitution rates are among the highest reported for any bacteria, and around two orders of magnitude higher compared with other inherited arthropod endosymbionts. The absence of mismatch repair loci *mutS* and *mutL* is conserved across *Spiroplasma *, and likely contributes to elevated substitution rates. Further, the closely related strains *s*Mel and *s*Hy (>99.5 % sequence identity in shared loci) show extensive structural genomic differences, which potentially indicates a higher degree of host adaptation in *s*Hy, a protective symbiont of *Drosophila hydei*. Finally, comparison across diverse *Spiroplasma* lineages confirms previous reports of dynamic evolution of toxins, and identifies loci similar to the male-killing toxin Spaid in several *Spiroplasma* lineages and other endosymbionts. Overall, our results highlight the peculiar nature of *Spiroplasma* genome evolution, which may explain unusual features of its evolutionary ecology.

## Data Summary

All novel sequencing data are available through National Center for Biotechnology Information (NCBI) repositories: raw reads for *s*Hy-Tx under BioProject accession PRJNA274591; raw reads for *s*Mel-Br under BioProject accession PRJNA507275; and all other raw reads and all assemblies under BioProject accession PRJNA640980.

Alignments for phylogenetic reconstructions, and a supplementary protocol are available from Zenodo under the DOI 10.5281/zenodo.3903209 (https://zenodo.org/record/3903209#.YBkwNzH7SM8).

Supplementary Material is available under the DOI 10.6084/m9.figshare.13584902.

All *Spiroplasma* genome assemblies used for comparative genomics are available from NCBI repositories, with the accession numbers listed in Table S1.

**Impact Statement**

Symbionts of arthropods are ubiquitous in nature and profoundly impact host biology. Most inherited bacterial symbionts of insects evade culturing outside their hosts, and as a consequence we have a limited understanding of evolutionary processes driving symbiont–host interactions. We studied *Spiroplasma poulsonii *, a naturally occurring symbiont of *Drosophila* that may act as male killer and protective symbiont. We monitored the evolution of the symbiont in the absence of selection for a host-associated strain and for a strain in a recently established axenic culture. Using whole-genome sequencing and re-sequencing, we observed rates of molecular evolution in *S. poulsonii* that are two orders of magnitude faster than in other microbes with similar ecology. Our findings explain repeatedly observed spontaneous loss of *Spiroplasma *-induced phenotypes and peculiar aspects of *Spiroplasma* evolutionary ecology. They further demonstrate that *Spiroplasma* is a unique model for the study of the genomic basis of symbiont adaptation to hosts.

## Introduction

Many bacterial lineages have evolved to become associates of animal hosts [1]. In arthropods, such associations are the rule, and maternally inherited, endosymbiotic bacteria are especially common and diverse [2, 3]. Prominent examples include obligate nutritional symbionts of blood and sap feeders [4], reproductive manipulators [5], and protective symbionts [6]. Collectively, inherited symbionts of arthropods are highly diverse with respect to potential benefits and costs induced, and their degree of adaptation to hosts. For example, even within a single lineage of inherited symbionts – *Wolbachia* – there are nutritional mutualists [7], protective symbionts [8] and reproductive manipulators [9]. Importantly, these symbiont-conferred traits may be modulated by environmental factors [10] and host genetic background [11].

*Spiroplasma* are small, helical bacteria that lack cell walls. Like other Mollicutes (* Mycoplasma *-like organisms), they are exclusively found in host association and may be pathogenic [12]. Among the first discovered spiroplasmas were plant pathogens (reviewed by Saglio and Whitcomb [13]) and an inherited symbiont of *Drosophila* with the ability to shift sex ratios towards females [14]. Subsequently, *Spiroplasma* symbionts were found in a number of different *Drosophila* species [15], as well as many other arthropods [16]. More recently, molecular evidence was brought forward for *Spiroplasma* symbionts in non-arthropod animals [17, 18]. In insects, two phenotypes induced by inherited *Spiroplasma* have been studied in detail (reviewed by Anbutsu and Fukatsu [19], and Ballinger and Perlman [20]). Firstly, *Spiroplasma* can enhance survival rates of its hosts under parasite or parasitoid attack. In *Drosophila neotestacea*, *Spiroplasma* inhibits growth and reproduction of the parasitic nematode *Howardula* and, thus, reverses nematode-induced sterility [21]. Further, *Spiroplasma* symbionts may greatly enhance *Drosophila* survival when attacked by parasitoids [22, 23]. Both protective phenotypes are mediated by *Spiroplasma *-encoded RIP (ribosome-inactivating protein) toxins, which target the attackers’ ribosomes [24–26]. These processes may be complementary to protection through competition between *Spiroplasma* and parasitoids for lipids in the host haemolymph [27]. Secondly, *Spiroplasma* kills males early in development in several species of *Drosophila*, planthoppers [28], ladybird beetles [29], lacewings [30] and butterflies [31]. The male-killing phenotype is also linked to a toxin: in *Drosophila melanogaster*, a *Spiroplasma* protein containing OTU (ovarian tumour-like deubiquitinase) and ankyrin domains was demonstrated to kill male embryos and, thus, termed *Spiroplasma poulsonii* androcidin (‘Spaid’) [32].

*Spiroplasma *-induced phenotypes have been commonly observed to be dynamic compared with other symbiont traits, with repeated observation of phenotype change within laboratory culture over relatively short time frames. For example, spontaneous loss of male killing was found in *Spiroplasma* symbionts of *Drosophila nebulosa* [33] and *Drosophila willistoni* [34], and spontaneous emergence of non-male-killing *Spiroplasma* in *D. melanogaster* has occurred at least twice in a single culture [32, 35]. In addition, *Spiroplasma* symbionts artificially transferred from their native host *Drosophila hydei* into *D. melanogaster* initially caused pathogenesis, but evolved to become benign over just a few host generations [36]. Genomic analysis has further documented dynamic *Spiroplasma* evolution driven by viral proliferation [37–39] and by extensive transfer of genetic material between plasmids, and from plasmids to chromosomes [40, 41]. Notably, plasmids in *Spiroplasma* commonly encode the systems that establish their phenotypic effect on their host [42]. Examples include Spaid [32] and RIP genes [26], and rapid plasmid evolution may, therefore, contribute to the high phenotypic evolvability of *Spiroplasma *. Furthermore, elevated rates of evolution were found in various *Mycoplasma* species [43, 44], which are pathogens closely related to *Spiroplasma* [45].

Bacteria adapting to hosts often follow similar evolutionary trajectories, and both increased mutational rates and proliferation of mobile genetic elements are commonly observed in diverse symbiotic taxa [46]. Therefore, spontaneous loss of *Spiroplasma* phenotypes and high genomic turnover could be explained by evolutionary mechanisms common to all symbiotic taxa. Although independent findings discussed above suggest that *Spiroplasma* symbionts may evolve quickly, this has never been quantified, and it has remained unclear how substitution rates compare to those of other symbionts. However, rates and patterns of evolutionary change may have important implications for *Spiroplasma* evolutionary ecology, and for its potential use in biological-control programmes [47, 48].

In this study, we systematically investigated rates and patterns of *Spiroplasma* evolution. To this end, we employed two strains of *S. poulsonii *. (i) *s*Hy is a natural associate of *D. hydei* [49] and confers protection against parasitic wasps [22]. We monitored its evolution over ~10 years in fly culture, and determined changes through *de novo* reference genome sequencing and re-sequencing of multiple isolates. (ii) *s*Mel occurs naturally in *D. melanogaster*, where it kills male offspring [50] and protects from parasitoids [23]. Using the previously established complete genome [51] and cell-free culture [52], we re-sequenced isolates spanning ~2.5 years of evolution. This approach enabled tracing *Spiroplasma* evolution on various levels: within-strain (<10 years divergence), between strains, and through comparison with other *Spiroplasma* genomes, across a whole clade of arthropod symbionts. It further allowed comparison between symbionts evolving in a host and in axenic culture. We observed that rates of molecular evolution and chromosomal rearrangements in *S. poulsonii* are substantially faster than in other inherited bacteria, and that this has resulted in markedly different genomic organization in *s*Hy and *s*Mel. Our work, thus, highlights *S. poulsonii* as an amenable, highly evolvable but potentially unpredictable model insect symbiont.

## Methods

### *Spiroplasma* strains and genome sequencing

**Fig. 1.**
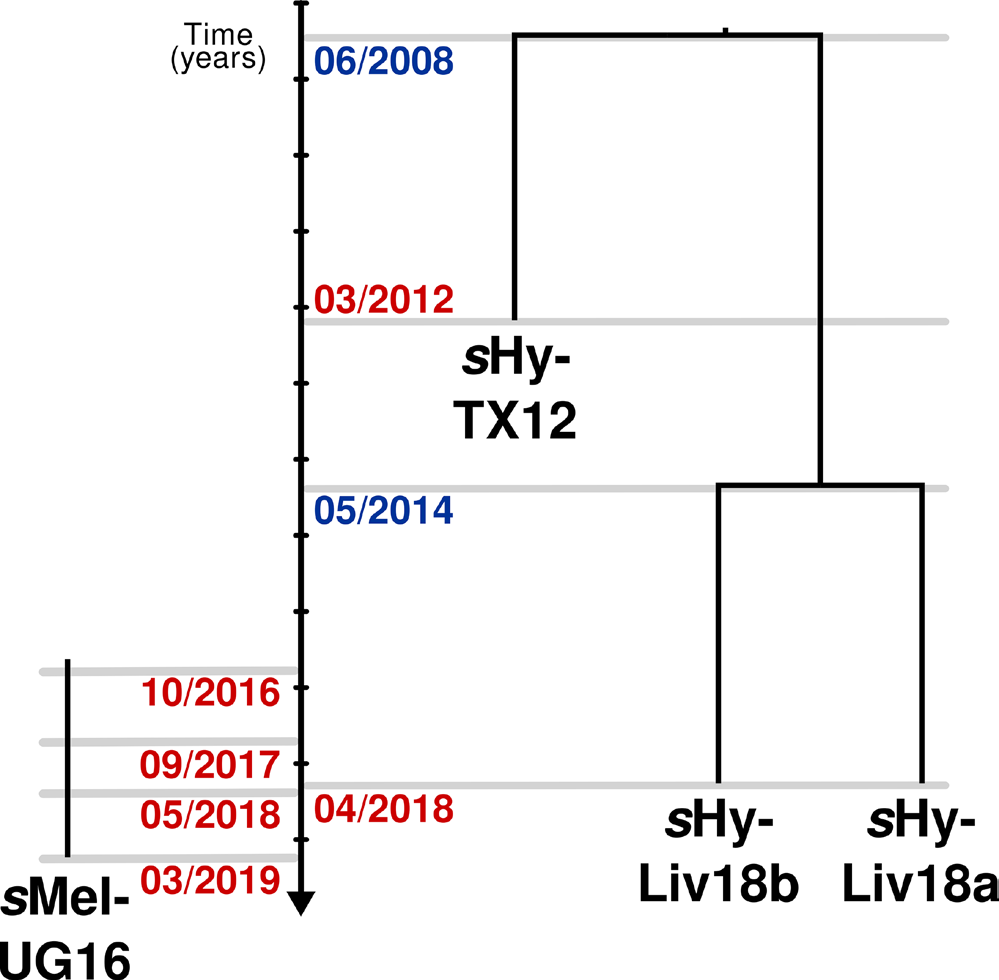
Overview on *S. poulsonii* strains investigated in this study. Three *s*Hy strains were sampled from *D. hydei* cultures over a total time span of 10 years. Blue dates denote splitting of *D. hydei* cultures, red dates mark *Spiroplasma* extraction for sequencing. For *s*Mel, samples from four points of a time series of axenic culture were sequenced (HL, start of culture; P14, after 14 passages; P57, after 57 passages; P79, after 79 passages). Here, red dates correspond to the date of isolation for sequencing.

We documented the evolution of the *S. poulsonii* strains *s*Hy and *s*Mel in fly hosts and in cell-free culture, respectively (Table 1). *s*Hy is one of the *Spiroplasma* symbionts naturally associated with *D. hydei* [53]. Our evolution experiments covered approximately 10 years and three variants of *s*Hy, all of which all originated from a single isolate (Fig. 1). Over the course of the study, *D. hydei* hosts were maintained on a standard corn meal agar diet (1 % agarose, 8.5 % sugar, 6 % maize meal, 2 % autolysed yeast, 0.25 % nipagin), at 25 °C, and a 12 h:12 h light:dark cycle. To isolate *Spiroplasma s*Hy-Liv DNA from *D. hydei*, we followed the protocol of Paredes *et al*. [51]. We collected approximately 300 *D. hydei* virgins and aged them for 4 weeks. The flies were then anesthetized with CO_2_ and pricked in the anterior sternopleurum using a sterile needle. Haemolymph was extracted via centrifugation (10 000 ***g***, 5 min, at 4 °C) through a 0.45 µm filter (Corning Costar Spin-X). To avoid haemolymph coagulation, we only extracted batches of 20 flies at a time and immediately transferred extracted haemolymph into ice-cold PBS solution (1× PBS buffer: 137 mmol NaCl, 2.7 mmol KCl, 10 mmol Na_2_HPO_4_, 1.8 mmol KH_2_PO_4_). Extracts of all batches were combined, and the PBS solution with *Spiroplasma* cells was centrifuged (12 000 ***g***, 5 min, at 4 °C). Supernatant PBS was discarded, and the cell pellet was subjected to DNA extraction using a phenol/chloroform protocol and subsequent ethanol precipitation. DNA was eluted in water, and sequenced at the Centre for Genomics Research at the University of Liverpool (UK). Libraries were prepared using a Nextera XT kit and sequenced as 2×250 bp runs on an Illumina MiSeq sequencer.

**Table 1.** *
S. poulsonii* strains investigated in this study Please note that we follow the naming scheme suggested by Ballinger and Perlman [26].

Name used in the current study	Natural host	Establishment in fly or cell-free culture	Source and date of DNA extraction	Also known as
***s*** **Hy**				
*s*Hy-Tx12	*Drosophila hydei*	01/2004 [53]	Haemolymph extract, 03/2012	Hap_1, TEN104-106
*s*Hy-Liv18a	*Drosophila hydei*	06/2008 (copy of *s*Hy-Tx12)	Haemolymph extract, 04/2018	–
*s*Hy-Liv18b	*Drosophila hydei*	03/2014 (copy of *s*Hy-Liv18A)	Haemolymph extract, 04/2018	–
***s*** **Mel**				
*s*Mel-Ug16	*Drosophila melanogaster*	10/2016 [52]	Cell-free culture, four time points in 2016–2018	MSRO-Uganda
*s*Mel-Br18	*Drosophila melanogaster*	07/1997 [54]	Whole fly, 03/2018	MSRO-RED42

DNA extraction for the *Spiroplasma* strain *s*Hy-Tx followed the protocol described above, with the following modifications: immediately after the piercing, 35–40 flies were placed into 0.5 ml microcentrifuge tubes previously pierced in the bottom, each of which was placed within a 1.5 ml microcentrifuge tube containing 20 µl PBS, and centrifuged at 4500 ***g*** for 10 s to collect haemolymph. DNA was recovered using a chloroform/ethanol procedure (Supplementary protocol 1) and diluted in AE buffer (Qiagen). A pair-ended library was constructed by Eureka Genomics. DNA was fragmented, end repaired, A′ tagged, ligated to adaptors, size-selected and enriched with 25 cycles of PCR during which an index was incorporated to the sample. Sample preparation was performed according to Illumina’s Multiplexing Sample Preparation Guide and Eureka Genomics’ proprietary method. The resulting library was subjected to Illumina HiSeq 2500 sequencing at the Texas A&M AgriLife Genomics and Bioinformatics Services Facility (College Station, TX, USA).

*s*Mel is a naturally occurring *Spiroplasma* symbiont of *D. melanogaster* best known for its male-killing phenotype [32, 54]. Cell-free culture of *s*Mel-Ug was recently accomplished [52], and we used a time series of this culture to investigate *s*Mel molecular rates of evolution outside of host tissues. The time series covered 29 months in total, and sequencing was performed at four time points (Fig. 1). Illumina DNA sequencing was performed at MicrobesNG using the ‘standard service’. Culturing, passaging and DNA extraction was performed as described by Masson *et al*. [52].

We further sequenced a single isolate of *s*Mel-Br deriving from a strain established in 1997 (Table 1). For Illumina sequencing, 1–2 g of whole body flies from *Spiroplasma *-positive *D. melanogaster* were collected for CTAB (*N*-cetyl-*N*,*N*,*N*-trimethylammonium bromide)/phenol based DNA extraction. Illumina libraries were prepared with an Illumina TruSeq DNA kit and sequenced on an Illumina NovaSeq-6000 S2 150 paired-end flow cell by Texas A&M AgriLife Genomics and Bioinformatics Services Facility. For Oxford Nanopore MinION sequencing, heads were first removed by immersing whole flies in liquid nitrogen and vigorous shaking on a metal sieve. The headless specimens were immediately subjected to phenol/chloroform extraction. Nanopore library preparation was performed using an Oxford Nanopore ligation sequencing kit 1D (SQK-LSK108), following precautionary measures to ensure long read lengths. The library was then sequenced on a MinION sequencing device (MIN-101B) with a SpotON Flow Cell Mark 1 (R9.5-FLO-MIN107.1). Base calling was performed using Albacore version 2.0 (Oxford Nanopore) and the reads were processed with Porechop version 0.24 [55] to remove adapter sequences. A draft assembly of *s*Mel-Br was created using Illumina and Nanopore reads and Unicycler version 0.4.7 [56]. The assembly was fragmented, but did contain three circular contigs with sequence similarity to other *Spiroplasma* contigs.

### *s*Hy reference genome sequencing and annotation

In order to reconstruct a high-quality reference genome for *s*Hy, we extracted DNA from 30 *D. hydei* virgin females carrying the symbiont. To limit the impact of potentially heterogeneous *Spiroplasma* populations within the flies, we used F3 individuals from a single isofemale line for DNA extraction. The flies were anesthetized at −20 °C, washed with 70 % ethanol and briefly dried. The flies were then homogenized in G2 lysis buffer from the Qiagen genomic DNA buffer set using a 1 ml dounce tissue grinder (DWK Life Science), and DNA was extracted from the homogenate using a Qiagen Genomic-Tip 20/G, following the protocol modifications from Miller *et al*. [57]. A total of 4 µg DNA was then used in the ‘1D gDNA long reads without BluePippin protocol’ library preparation protocol with a 1D ligation sequencing kit (SQK-LSK108; Oxford Nanopore). Sequencing was performed on a MinION sequencer (Oxford Nanopore) using a single FLO-MIN106 R9 flow cell for 48 h, and the raw Nanopore signals were base-called using Albacore version 2.3.1.

All reads passing the quality checks were used to create an assembly with unicycler version 0.4.7 [56]. The resulting assembly contained six circular contigs (~15 kb –~1.6 Mbp) with high similarity to the previously published *s*Mel genome as determined by blast+ version 2.9 searches [58] in Bandage [59]. We also created a hybrid assembly with Unicycler using all long reads and the *s*Hy-Liv18a Illumina reads. The hybrid assembly was more fragmented, but did contain three contigs that spanned almost the entire 1.6 Mbp circular contig of the long-read-only assembly. Because hybrid assemblies are typically more accurate than assemblies based on long reads only [60], we corrected the long-read assembly using the hybrid contigs. This was achieved by calling differences between the assemblies with the ‘dnadiff’ function implemented in mummer version 3.18 [61], and correcting the assembly accordingly. Finally, pilon version 1.23 [62] was used to polish the corrected assembly, again using the *s*Hy-Liv18a Illumina reads. After nine rounds of polishing with pilon, no further improvements could be observed and the assembly was considered final.

### Rates of molecular evolution in *s*Hy and *s*Mel

We determined changes in *s*Hy and *s*Mel chromosomes over time by employing the snippy pipeline version 4.1.0 [63]. We used our newly assembled *s*Hy genome and the previously sequenced *s*Mel genome [52] as references, annotated using the prokka pipeline version 1.13 with standard parameters and the *Spiroplasma* genetic code (translation table 4) [64]. *s*Hy and *s*Mel variants determined by snippy were filtered to only include positions that were covered by all *s*Hy or all *s*Mel Illumina libraries with at least 5× coverage, respectively. Further, to compare rates of molecular evolution between *Spiroplasma* and other microbial species, we counted the number of changes in third codon positions of coding sequences (CDSs). For both strains, we only considered CDSs that were entirely covered by a sequencing depth of at least 5× in all Illumina libraries (see Table S2 for sequencing and mapping statistics). Rates of molecular evolution were calculated by number of observed changes at third codon positions/number of considered third codon positions/time in years over which the changes were observed. For *s*Hy, using *s*Hy-Liv18a as a reference, we calculated two rate estimates, one based on comparison with *s*Hy-Liv18b, and one by comparing *s*Hy-Tx12 to the reference (Fig. 1). For *s*Mel, the haemolymph extract from which the culture was established was used as the reference (‘HL’; Fig. 1), and three rate estimates were calculated based on the changes observed in the three sampled time points of the culture (Fig. 1).

### Comparative genomics

We first compared the newly assembled *s*Hy genome with the most closely related, fully sequenced *Spiroplasma* genome, *s*Mel. Both genomes were annotated using the Kyoto Encyclopedia of Genes and Genomes (KEGG) and BlastKOALA [65, 66], and orthologous protein sequences determined using orthofinder version 2.2.3 [67]. Synteny between the genomes was assessed through aligning the genomes with minimap2 version 2.15 [68]. Genome degradation was investigated by documenting the number of unique KEGG numbers for *s*Hy and *s*Mel, all of which were verified by blast+ searches. For each strain, we considered genes to be likely pseudogenized or truncated if the longest CDS within an orthogroup spanned at most 60 % of the CDS length from the other strain.

Secondly, we compared *s*Hy with 17 *Spiroplasma* genomes from the Citri–Chrysopicola–Mirum clade (see Table S1 for a full list of names and accession numbers). All genomes were annotated using prokka version 1.4.0, and orthology between predicted CDSs was established using orthofinder. We extracted single-copy orthologues present in all strains, and aligned each locus separately with the L-INS-i method implemented in mafft version 7.450 [69]. Recombination was evaluated with pairwise homoplasy index and window sizes of 100, 50 and 20 amino acids for each locus [70], and any locus showing evidence for recombination was excluded. We used iq-tree version 1.6.10 [71] to reconstruct a core-genome phylogeny from the remaining 96 loci (covering 26 019 amino acid positions). Each locus was treated as a separate partition with a distinct evolutionary rate [72], and optimal models and number of partitions were estimated with iq-tree [73, 74]. Branch support was assessed using 1000 replicates of ultrafast bootstraps [75], and approximate likelihood ratio test [76] in iq-tree.

For all genomes, insertion sequence elements were annotated with prokka. Prophage regions were predicted with phispy version 3.7.8, which uses typical prophage features (e.g. gene length, strand directionality, AT/GC skews, insertion points) for its predictions [77]. We also used the phaster web server, which uses sequence similarities and other criteria to predict prophages [78]. We screened for the presence of toxin genes implied in protective phenotypes [25, 26] and male killing [32]. To this end, we downloaded UniProt sequence alignments from the Pfam database [79] for the protein families RIP (PF00161) and OTU (PF02338, OTU-like cysteine protease), respectively. We used these alignments as databases for searches with hmmer version 3.2.1 [80], and protein sequences from all genomes as queries. Domain architecture of all matching proteins was then determined using PfamScan with an *E* value of 0.001 [81], signalp 5.0 [82] and tmhmm 2.0 [83]. RIP domains showed a high degree of divergence and, therefore, were aligned to the reference RIP HMM (hidden Markov model) profile using hmmalign from the hmmer software package. From this alignment, positions present in fewer than three sequences were removed, and a phylogeny reconstructed with iq-tree. OTU domains were aligned using mafft, and a phylogeny reconstructed with iq-tree.

Thirdly, we determined plasmid synteny across *S. poulsonii* strains. In addition to the plasmids of *s*Mel and *s*Hy, we also included a plasmid of *s*Neo and all circular contigs from our *s*Mel-Br assembly. Plasmids were annotated using prokka version 1.13 with a protein database composed of all plasmid proteins available from the National Center for Biotechnology Information (NCBI) GenBank. Plasmid alignment and visualization were performed with alitv version 1.06 [84].

For an extended set of 31 *Spiroplasma* genomes that also included strains from the Apis clade (Table S1), we performed gene tree–species tree reconciliations to investigate the evolutionary history of prophage loci. To this end, we annotated all genomes and determined orthologous groups of loci as described above. Orthogroups were blasted against a database containing all available *Spiroplasma* virus proteins from NCBI Protein (*N*=192). Genes were classified as ‘prophage related’ if hits were at least 60 % identical over 50 % of the length of any viral protein. Reconciliation was performed with the prophage loci using generax version 1.2.2, using maximum-likelihood gene trees determined with iq-tree as starting trees and the LG+G model for all loci. We predicted CRISPR/Cas systems and CRISPR arrays using the tools crisprcastyper version 1.2.1 [85] and crispridentify [86], respectively.

### Data visualization

Figures were prepared in R version 3.6.2 [87] using the packages ‘ape’ [88], ‘cowplot’ [89], ‘ggalluvial’ [90], ‘ggplot2’ [91], ‘ggtree’ [92] and ‘ggridges’ [93]. Phylogenetic trees and domain architecture of toxin loci were visualized with evolview version 3.0 [94].

## Results

### Rates and patterns of evolutionary change in *S. poulsonii*

The *S. poulsonii s*Hy reference genome comprises a single circular chromosome (1 625 797 bp) and five circular contigs (23 069–15 710 bp) with sequence similarities to plasmids from other *Spiroplasma *. The chromosome contains 1584 predicted CDSs, a single rRNA cluster and 30 tRNAs. Overall, genome content and metabolic capacities are highly similar to the previously sequenced, very closely related *s*Mel genome [51], and a detailed comparison follows further below.

**Fig. 2.**
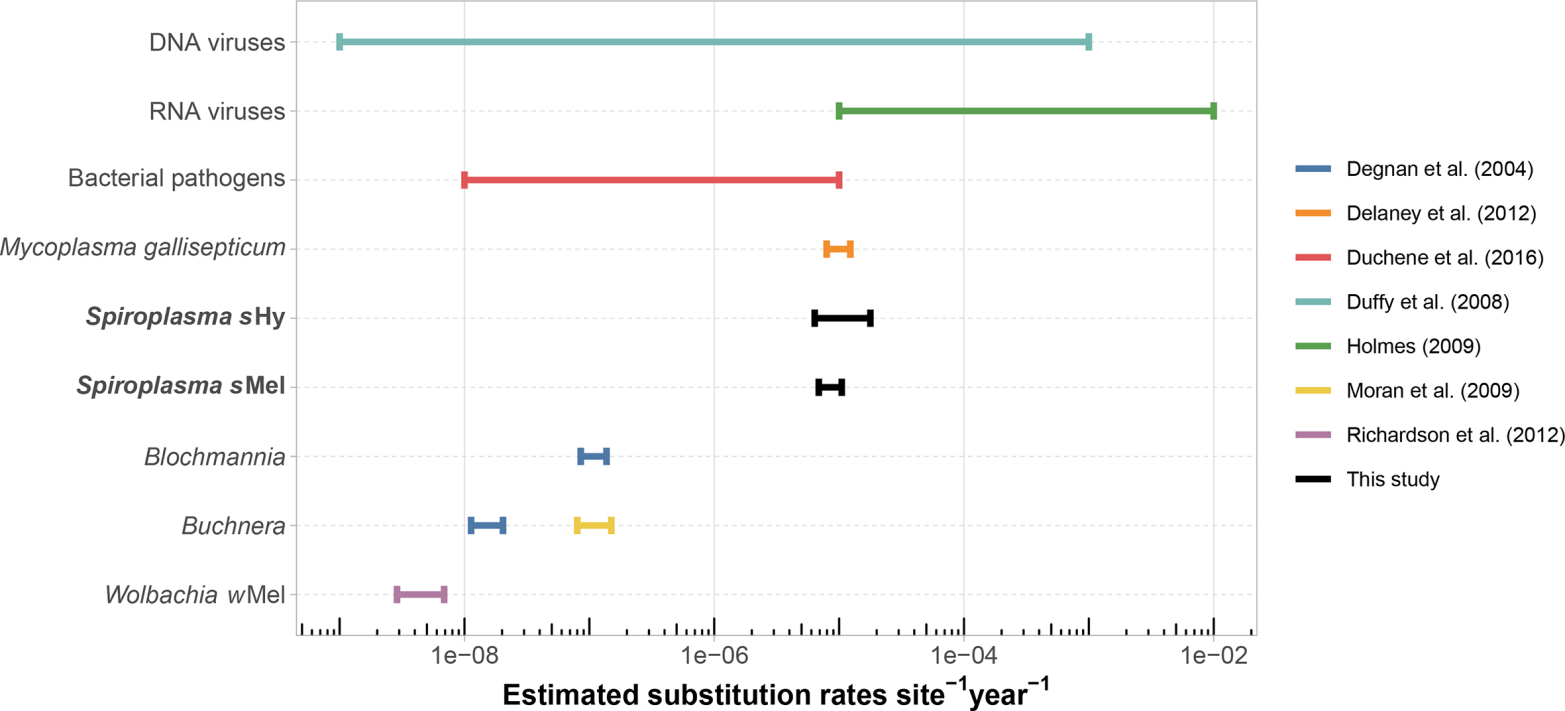
Comparison of estimated evolutionary rates across various microbes. Estimates obtained in this study are highlighted in bold. All bacterial rates are from chromosomal sequences only. DNA viruses – estimates as summarized by Duffy *et al*. [141 ], including ssDNA viruses and dsDNA viruses. RNA viruses – range of RNA virus substitution rates from Holmes [97 ]. Bacterial pathogens – approximate range of evolutionary rates estimated from genome-wide data of 16 bacterial pathogens (* Acinetobacter baumannii *, *Bordetella pertussis *, *Enterococcus faecium *, *Klebsiella pneumoniae *, *Mycobacterium leprae *, *Mycobacterium tuberculosis *, *Neisseria meningitidis *, *Pseudomonas aeruginosa *, *Salmonella enterica *, *Shigella dysenteriae *, *Shigella sonnei *, *Staphylococcus aureus *, *Streptococcus pneumoniae *, *Streptococcus pyogenes *, *Vibrio cholerae *, *Yersinia pestis *) as determined by Duchêne *et al*. [96 ]. *Mycoplasma gallisepticum* – genome-wide rate estimated from multiple isolates over a 12 year period [44 ]. *Blochmannia* and *Buchnera* (blue line) – based on 16S rDNA, *gidA* and *groEL* sequences, taken from Degnan *et al*. [142 ]. *Buchnera* (yellow line) – genome-wide rate estimated from seven isolates [102 ]. *Wolbachia w*Mel – genome-wide data extracted from 179 *D. melanogaster* SRA libraries [108 ].

To estimate rates of molecular evolution in *S. poulsonii *, we measured chromosome-wide changes in CDSs of *Spiroplasma* from fly hosts (*s*Hy) and axenic culture (*s*Mel) over time. Our estimates for *s*Hy and *s*Mel are overlapping and range from 6.4×10^−6^ – 1.8×10^−5^ changes per position per year. This rate exceeds the rate reported for other symbiotic bacteria such as *Wolbachia* and *Buchnera* by at least two orders of magnitude (Fig. 2). Our estimate overlaps with rates calculated from some fast-evolving human pathogens (e.g. *Enterococcus faecium* and *Acinetobacter baumannii *), and with evolutionary rates observed in the poultry pathogen *Mycoplasma gallisepticum* (which is closely related to *Spiroplasma *). Indeed, *S. poulsonii* substitution rates fall at around the lower estimates for RNA viruses (Fig. 2).

**Fig. 3.**
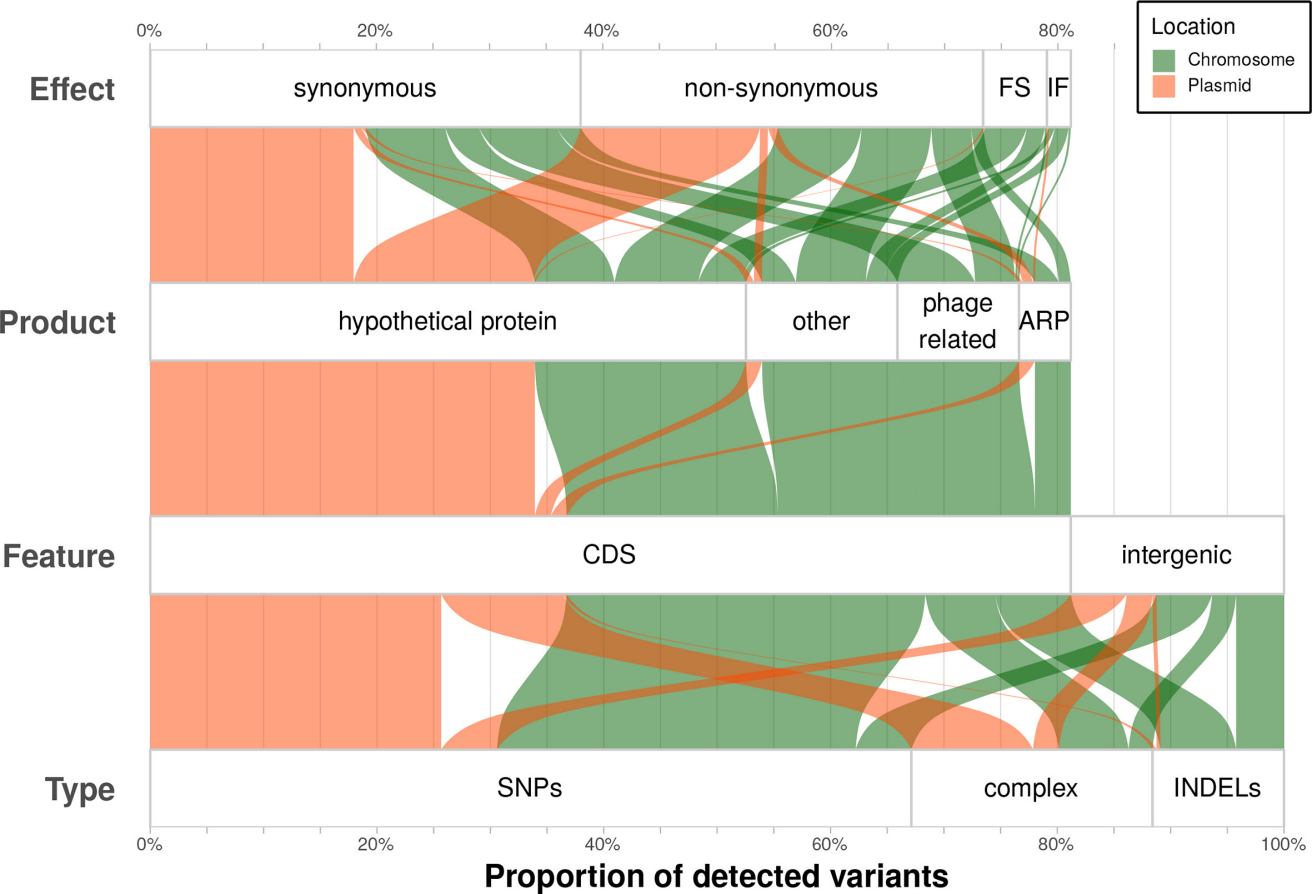
Overview of all observed variants in *s*Hy (100 %=570 variants). All annotations (type, feature, product, effect) are taken from results of the snippy pipeline. ARP, Adhesion-related protein; complex, variants that span multiple nucleotides and/or SNPs; FS, frame shift; IF, in frame; INDELs, insertions and deletions.

Over ~10 years of evolution, we observed a similar absolute number of variants on the chromosome (~1.6 Mbp) and plasmids (~0.1 Mbp) of *s*Hy, i.e. relatively more changes on the plasmids (Fig. 3, Table S3). Most variants in CDSs affected hypothetical proteins, but were enriched in prophage-associated loci and adhesin-related proteins (Fig. 3). Overall, *s*Hy variants in CDSs were about equally often found to be synonymous as non-synonymous (Fig. 3), and changes were biased towards GC >AT substitutions (Fig. S1). The changes in *s*Mel over ~2.5 years in culture affected only 15 different CDSs in total, of which 4 were adhesin-related proteins and 3 lipoproteins (Table S3). Thus, the rates and patterns of evolutionary change are similar between the axenically cultured *s*Mel and the host-associated *s*Hy.

**Fig. 4.**
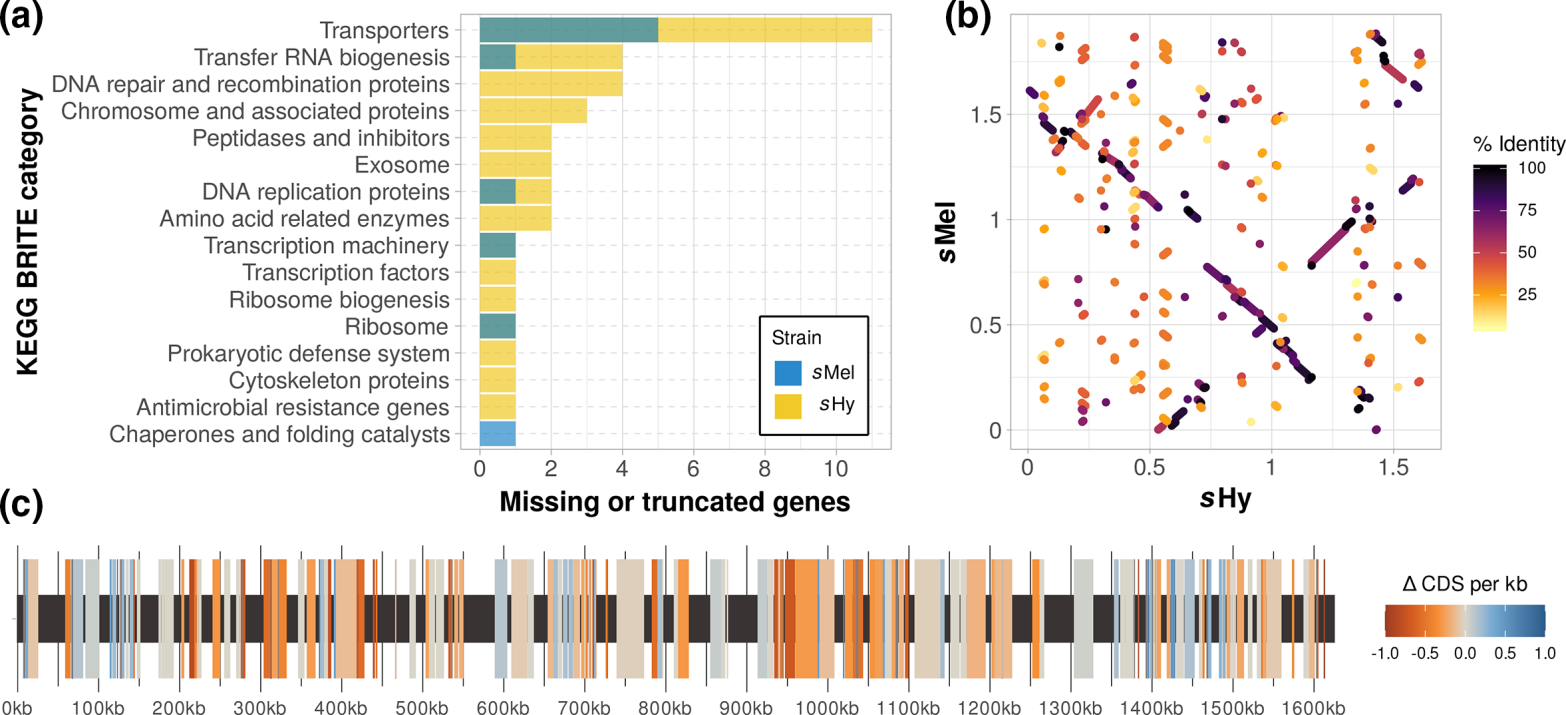
Comparison of genomic features between *s*Hy and *s*Mel. (a) Number of genes with KEGG annotation that are present in one strain, but absent or truncated in the other. See Table S4 for a complete list. (b) Synteny as determined with minimap2. Syntenic blocks are coloured by sequence similarity. (c) Genomic map of *S. poulsonii s*Hy with blocks syntenic to *s*Mel highlighted. The mean number of predicted CDSs kb^−1^ in these blocks is displayed as difference between *s*Hy and *s*Mel: positive and negative values indicate fewer CDSs in *s*Hy or *s*Mel, respectively. Syntenic blocks were determined with a blast+ search using the *s*Mel chromosome sequence as query against a *s*Hy chromosome database, keeping a single best match for any *s*Hy region, and discarding hits below 1000 bp and under 95 % nucleotide sequence similarity. Out of 635 syntenic blocks, 324 (spanning 852 730 bp) contained fewer CDSs in *s*Hy, and 164 (345 546 bp) contained more CDSs in *s*Hy, with 147 regions (334 084 bp) showing no difference in CDS number.

Comparing the genomes of *s*Hy and *s*Mel revealed a notable contrast between the high degree of nucleotide sequence identity on the one hand, and striking structural and gene content differences on the other hand. In 604 single copy orthologues shared between the genomes, the mean nucleotide identity was >99.5 %. However, compared with *s*Mel, *s*Hy is reduced in gene content (1584 vs 2388 genes), chromosome size (1.61 vs 1.88 Mbp) and coding density (65 vs 82 %). Further, *s*Hy contains fewer predicted insertion sequences (9 vs 89 in *s*Mel) and intact prophages as predicted with phaster (*s*Mel, 16 such regions spanning 426 kb; *s*Hy, 2 regions, 14 kb), but not with phispy (Fig. S2). Both *s*Mel and *s*Hy have a number of missing or truncated (i.e. potentially pseudogenized) genes when compared with each other, but the level of genomic deterioration is higher in *s*Hy, and covers a range of different genes (Fig. 4a, Table S4). Notable pseudogenized or absent loci in *s*Hy include parts of the phosphotransferase system, in particular the loci required for the uptake of *N*-acetylmuramic acid and *N*-acetylglucosamine (see Table S3 for a full list). Further, while *recA* is truncated in *s*Mel, the copy in *s*Hy appears complete and functional. As suggested by Paredes *et al*. [51], the loss of *recA* function in *s*Mel is, therefore, likely very recent.

Strikingly, there is evidence for a history of extensive chromosomal rearrangements since the last common ancestor of *s*Hy and *s*Mel, and genome-wide synteny between the strains is low (Fig. 4b). On average, syntenic blocks between the strains contained fewer predicted CDSs for *s*Hy (on average −0.3 CDSs kb^−1^; Fig. 4c), in line with a higher degree of genomic deterioration in *s*Hy compared with *s*Mel. A comparison of plasmids across different *S. poulsonii* strains (*s*Hy, *s*Mel-Ug, *s*Mel-Br and *s*Neo) revealed similar gene content across the plasmids of different strains, but large differences in arrangement and number (Fig. S3).

### Genome and toxin evolution in the genus *Spiroplasma*

**Fig. 5.**
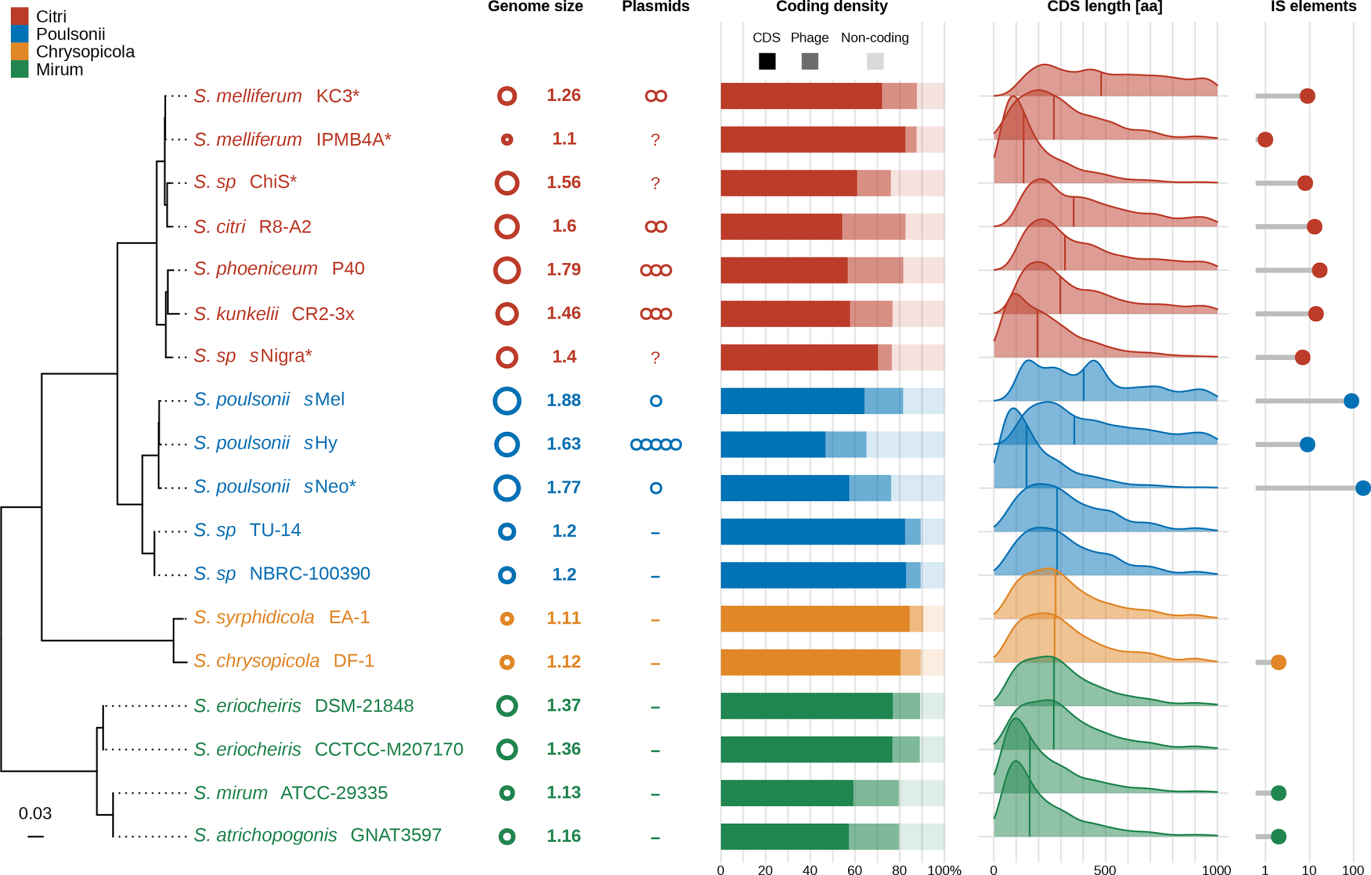
Genome properties of *Spiroplasma* strains from Citri, Poulsonii, Chrysopicola and Mirum clades. Genome size (Mbp) and plasmid number were taken from the literature (Table S1). Coding density, CDS length and number of IS elements were determined with Prokka, and proportion of prophage was estimated with PhiSpy. *Spiroplasma* phylogeny is based on partitioned maximum-likelihood analysis of 96 concatenated single-copy genes present in all of the genomes (26 019 amino acid positions). Scale bar correponds to inferred subsitutions per site. All nodes were maximally supported by UFB and aLRT. Asterisks indicate strains with genomes with draft status (i.e. a chromosome assembly split into multiple contigs).

Comparing *s*Hy with other sequenced strains of the *Spiroplasma* clades Citri, Poulsonii, Chrysopicola and Mirum showed dynamic genome evolution within this genus of symbionts (Fig. 5). All investigated spiroplasmas have reduced genomes (~1.1–1.9 Mbp), and the *S. poulsonii* strains *s*Hy, *s*Mel and *s*Neo are among the strains with the largest main chromosomes (Fig. 5). Plasmid numbers range from 0 to 5, and *s*Hy has the highest number of plasmids of any of the investigated strains, although plasmid number is unclear for some of the genomes with draft status, and *Spiroplasma citri* may have seven plasmids [95]. Prophage regions of varying sizes were predicted in all of the genomes (despite the lack of clear homologues to viral sequences in some of these) [39]. Reconciliation of prophage gene trees with the *Spiroplasma* species tree revealed that prophage proliferation has likely happened relatively recently, and repeatedly in the Citri and Poulsonii clades (Fig. S4). Prophage loci are entirely absent, or very low in number, for *Spiroplasma* strains that harbour CRISPR/Cas systems, or remnants thereof (Fig. S4). Further, *Spiroplasma* genome size correlated with the number of insertion sequences (Figs 5 and S5). The distribution of CDS lengths varies across the investigated genomes (Fig. 5), which may be explained by differences in proportion of prophage regions, level of pseudogenization and assembly quality. Overall, our comparison indicates that *s*Hy may be more degraded than its closest relatives *s*Neo and *s*Mel, with a smaller chromosome size, fewer predicted CDSs and mobile genetic elements, and the lowest coding density of these three (Fig. 5).

Spiroplasmas’ most striking phenotypes in insects have been mechanistically linked to toxin genes. For example, RIPs may protect *Spiroplasma* hosts by cleaving ribosomal RNA of parasites and parasitoids [25]. Five RIP loci are present in *s*Mel (RIP1–5, of which 3–5 are almost identical copies). As expected, the protective *s*Hy also encodes RIPs, but only has a single orthologue for RIP1. However, it contains an additional RIP gene in three copies that appears to be absent in *s*Mel, and one of these copies also contains ankyrin repeats (Fig. S6).

**Fig. 6.**
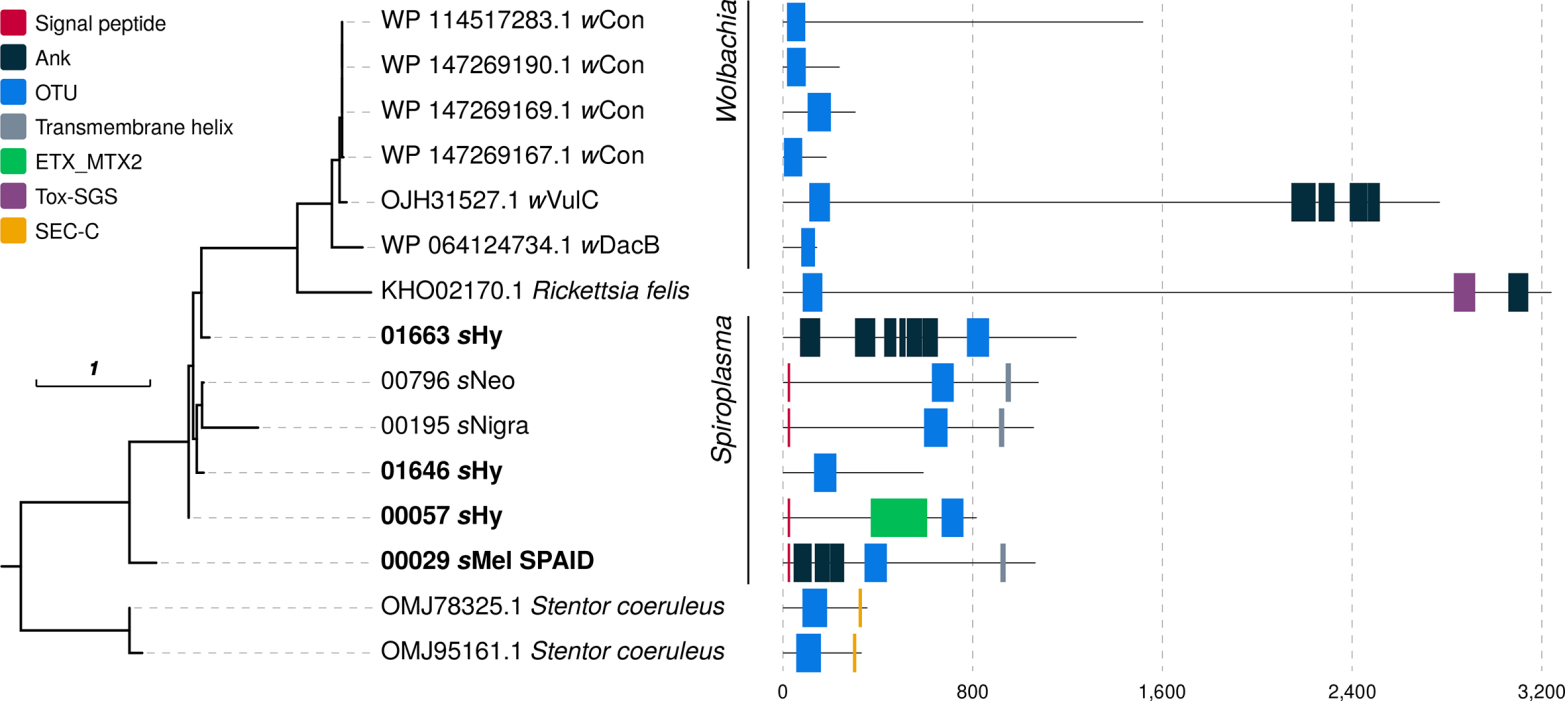
Spaid-like proteins in *Spiroplasma* and other symbionts. The maximum-likelihood tree was reconstructed from an alignment of OTU domains (147 amino acid positions), and domain predictions are based on pfamscan, signalp and tmhmm. Scale bar correponds to inferred subsitutions per site. Ank, Ankyrin repeats; OTU, ovarian tumour-like deubiquitinase; ETX_MTX2, *Clostridium* epsilon toxin ETX/* Bacillus* mosquitocidal toxin MTX2; Tox-SGS, salivary gland secreted protein domain toxin; SEC-C, SEC-C nucleic acid binding domain.

Further, the male-killing phenotype of *s*Mel was recently established to be caused by *Spiroplasma* androcidin (Spaid), and both ankyrin repeats and a deubiquitinase domain (OTU) of this gene are necessary to induce male killing [32]. hmmer searches using OTU domain profiles revealed a number of *Spiroplasma* loci similar to Spaid (Fig. 6) that, however, lack its characteristic domain composition. For example, *s*Hy encodes three loci similar to Spaid: one lacks a signal peptide, one has no ankyrin repeats and another encodes an epsilon-toxin-like domain in addition to the OTU domain (Fig. 6). Other bacterial loci with notable similarities to the *Spiroplasma* OTU could only be detected in the symbiotic taxa *Rickettsia* and *Wolbachia *. In the phylogenetic reconstruction of OTU domains rooted with the eukaryotic sequences (from the ciliate *Stentor coeruleus*), *Rickettsia* and *Wolbachia* loci are nested within the *Spiroplasma* sequences. This topology suggests lateral gene transfer of OTU-domain-containing proteins from *Spiroplasma* to the other intracellular taxa, although differences in genetic code between *Spiroplasma* and other bacteria likely limit the probability for transfer of functional protein-encoding genes.

## Discussion

### *Spiroplasma*, an exceptionally fast evolving symbiont

The substitution rates we observed in *S. poulsonii* are among the highest reported for any bacteria (Fig. 1). In a study comparing genome-wide evolutionary rates in bacterial human pathogens [96], most taxa showed considerably slower rates, and our estimate of *S. poulsonii* evolutionary rates overlaps with substitution rates of RNA viruses [97]. A number of reasons have been proposed for elevated substitution rates in bacterial symbionts and pathogens with small genome sizes. Firstly, host-associated microbes may acquire essential metabolic intermediates from their hosts and, thus, need not synthesize them [98]. Obsolete metabolic genes subsequently accumulate now neutral substitutions, resulting in pseudogenization. Secondly, reduced population sizes of symbionts, together with bottlenecks at transmission events, limit purifying selection of deleterious mutations [99] and mobile genetic elements [100] – both in turn create further genomic deterioration. Thirdly, genomes of host-associated microbes are AT rich [101], which makes introduction of errors through replication slippage more likely [102]. Fourthly, many symbionts lack DNA repair genes [103], which may result in increased substitution rates.

**Fig. 7.**
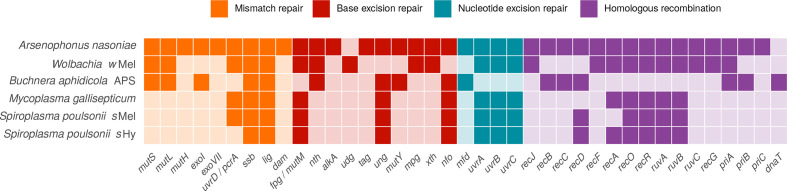
Absence (light colours) and presence (dark colours) of genes involved in DNA repair in *Buchnera aphidicola *, *Wolbachia w*Mel, *Mycoplasma gallisepticum *, and *S. poulsonii s*Hy and *s*Mel. *Arsenophonus nasoniae* is included as an example of a symbiont with a relatively large genome and complete DNA repair pathways. Genes involved in more than one pathway are listed only once, and those missing in all of the taxa are not displayed. Data is taken from the KEGG pathway database [66], and annotation of *s*Mel and *s*Hy genomes is as described in Methods.

All of these points very likely apply for *S. poulsonii *: its metabolic capacities are reduced compared with free-living microbes, it has a high proportion of pseudogenes and prophages, very high AT content, and lacks several DNA repair genes. However, many symbiotic bacteria show similar trends of genome evolution [104] and it is, therefore, somewhat puzzling that *S. poulsonii* stands out with this exceptionally high substitution rate. Loss of DNA repair genes has been shown to co-occur with elevated mutation rates in intracellular bacteria [105–107], and may explain our observations. The mismatch repair protein-encoding loci *mutS* and *mutL* are universally lacking in *Spiroplasma *, but are present in the slower-evolving symbionts *Wolbachia w*Mel and *Buchnera aphidicola* (Fig. 7) [108–110]. In *Escherichia coli *, hypermutator strains that have lost such loci have a ~10–300-fold increase of spontaneous mutation rates [111, 112], which is comparable to the approximately two orders of magnitude difference between substitution rates of *Buchnera* and *Spiroplasma* (Fig. 2). Like *Spiroplasma *, *Mycoplasma* universally lacks the mismatch repair loci *mutS*, *mutL* and *mutH* [113, 114]. In line with this observation, elevated evolutionary rates were hypothesized for mycoplasmas [43], and in our comparison, *Mycoplasma gallisepticum* appears to be the only bacterial taxon with substitution rates similar to *S. poulsonii* [44]. Absence of the DNA mismatch repair pathway may, thus, be ancestral to Entomoplasmatales (Spiroplasmataceae + Entomoplasmataceae) and contribute to the dynamic genome evolution across this taxon [115, 116]. Alternatively, increased substitutional rates caused by the loss of these loci could have arisen multiple times independently in Entomoplasmatales.

Further to absence of DNA repair genes causing elevated mutation rates, a recent comparative study demonstrated a strong negative correlation between mutation rate and genome size in free-living and endosymbiotic bacteria [117]. This correlation is, however, not apparent in the genomes of endosymbionts we have investigated. For example, the considerably slower evolving *Buchnera* genomes are much smaller than *Spiroplasma *, and *Wolbachia* would be predicted to have much larger genomes if their size were mainly determined by mutational rates. This suggests that substitution rates alone are a poor predictor for the sizes of genomes investigated here. Likely, these genome sizes result from an interplay of multiple factors such as population size, patterns of DNA repair gene absence and mutational rates [118, 119].

Our rate estimate is potentially biased by at least two factors. Firstly, we have only investigated laboratory populations of *S. poulsonii *. Each vertical transmission event creates symbiont population bottlenecks potentially increasing genetic drift and, thus, substitution rates [46]. Because the number of generations in natural populations of the *Spiroplasma* host *D. hydei* is lower compared with laboratory reared hosts, vertical transmission events are rarer under natural conditions and, therefore, substitution rates potentially lower. Further, laboratory strains could experience relaxed selection compared with natural symbiont populations. This may lead to higher substitution rate estimates from laboratory populations compared with natural populations. Secondly, substitution rates often appear larger when estimated over brief time periods [120]. Duchene *et al*. found that substitution rates measured over 10 years can be up to one order of magnitude larger than those measured over 100 years [96]. In agreement with such bias, we found more variants in our *s*Mel culture after 19 months than for the 29 months isolate (Table S3). The back mutations that likely have happened between 19 and 29 months of our *s*Mel culture would go unnoticed when comparing genomes over larger time scales.

More generally, it is difficult to estimate divergence times between *Spiroplasma* strains. The substitution rates estimated in *s*Hy and *s*Mel would suggest the two strains have diverged 1120–2260 years ago. However, this estimate is unreliable due to a number of factors that we cannot control for. For example, the number of generations per year for *Drosophila* hosts of *Spiroplasma* differ depending on species and location, and is expected to be lower in the wild compared with laboratory strains. Further, *Spiroplasma* may move between species, for example via ectoparasitic mites [121]. A number of partially sympatric *Drosophila* species carry similar *Spiroplasma* strains (*D. hydei*, *D. melanogaster*, *D. willistoni*, *D. neotestacea*) [15], and with our data it is impossible to determine the number and direction of potential *Spiroplasma* transfers between these species.

### Reductive genome evolution in *Spiroplasma*

According to a widely supported model of genome reduction in symbiotic bacteria [46], the first stages of host restriction involve accumulation of pseudogenes and mobile genetic elements, chromosomal rearrangements, increased substitution rates, and excess deletions. Advanced stages of host association are accompanied by further genome shrinkage, and purging of mobile genetic elements, which overall result in more stable chromosomes. Using these characteristics, different levels of genome reduction are apparent in the investigated *Spiroplasma* genomes, across which genome sizes correlate positively with number of mobile genetic elements (plasmids, prophages, insertion sequences; Figs 5 and S5). On this spectrum, *Spiroplasma syrphidicola* and *Spiroplasma chrysopicola* have the most reduced genomes, without homologues of prophage loci, and high levels of synteny between the two strains [39]. Therefore, it was argued that phages have likely invaded *Spiroplasma* only after the split of the Syrphidicola and Citri+Poulsonii clades [39]. Our prophage gene tree-species tree reconciliations are in line with this hypothesis, but also indicate that prophage proliferation has largely happened independently in different *Spiroplasma* lineages (Fig. S4). CRISPR/Cas systems have multiple origins in *Spiroplasma* [122] and only occur in strains lacking prophages (Fig. S4). While the absence of antiviral systems often coincides with prophage proliferation (e.g. in the Citri clade), several strains with compact, streamlined genomes lack CRISPR/Cas and prophages (e.g. TU-14 and NBRC-100390; Fig. S4). These strains also show other hallmarks of reduced symbiont genomes (small size, high coding density, lack of plasmids and transposons; Fig. 5), which is in line with the model of genome reduction discussed above and suggests prophage regions were purged from these genomes. Alternatively, these strains may never have been exposed to phages.

Using the model of genome reduction during restriction to the host environment introduced above, *S. poulsonii *’s genome characteristics suggest that such restriction has happened relatively recently. Although very closely related (99.5 % sequence identity) and found in very similar hosts, *s*Mel and *s*Hy differ markedly in coding density, genome size and proportion of prophage regions. Interestingly, phaster predicted 426 kb of intact phage regions in *s*Mel, but only 14 kb for *s*Hy (Fig. S2). Both phage proliferation in *s*Mel and prophage loss in *s*Hy could have contributed to this. However, when using the similarity agnostic tool phispy, the predicted prophage regions were similar in size between *s*Hy and *s*Mel (Fig. S2). This observation is compatible with degradation and/or pseudogenization of prophage regions in *s*Hy, which would lead to reduced sequence similarity to viral loci (and, thus, reduced detectability by phaster), but not entirely blur prophage characteristics employed by PhiSpy (e.g. gene length, strand directionality, AT/GC skews, insertion points) [77]. Since the split of the lineages, *s*Hy has not only lost prophage regions and insertion sequences compared with *s*Mel, but also several genes that are often lost in host-restricted bacteria, such as parts of the phosphotransferase system for the uptake of carbohydrates, one tRNA locus, and *uvrD*, which plays a role in mismatch repair and nucleotide excision repair (Table S3).

Using signatures of genomic degradation as a proxy, our findings collectively suggest that *s*Hy is in a more advanced stage of host restriction than *s*Mel. This may indicate co-adaptation with the host as a result of the fitness benefits associated with *s*Hy under parasitoid pressure, and the absence of detectable costs for carrying *s*Hy in *D. hydei* [22, 123, 124]. However, the *Spiroplasma* symbiont of *D. neotestacea*, *s*Neo, is also protective, does not cause obvious fitness costs [21], but has a less reduced genome (Fig. 5) [26]. Further, it is also possible that genome reduction in *s*Hy was mainly driven by stochastic effects or even by adaptation to laboratory conditions, as we have not investigated contemporary *s*Hy from wild *D. hydei* populations.

### Implications for *Spiroplasma* evolutionary ecology

*Spiroplasma* evolutionary ecology shows several parallels to that of the most widely distributed arthropod symbiont, *Wolbachia *: both symbionts are found in a range of different hosts [3], have the ability to invade novel hosts [125, 126], may confer protection [8, 22] but also kill males [127], and can spread across host populations swiftly [128, 129]. However, there are also pronounced differences between the symbionts: *Spiroplasma* rarely reaches the high infection frequencies often observed in *Wolbachia* [130, 131], and is arguably found in a more diverse host range that encompasses arthropods and plants [132], molluscs [18], echinoderms [17] and cnidarians [133]. Further, *Wolbachia* show greatly reduced spontaneous mutation rates compared with *Spiroplasma *, likely caused by a more complete set of DNA repair genes (Fig. 7).

In theory, fast evolutionary rates should enable *Spiroplasma* to adapt to novel hosts quickly (i.e. to reduce pathogenicity, and to maximize vertical transmission efficiency), and experimental studies have found high horizontal transmission efficiency of *Spiroplasma* [36, 134, 135]. Consistent with this, we found that genes implicated in host–symbiont compatibility and virulence have evolved especially fast in our evolution experiments. For example, adhesion-related proteins are important in cell invasion in other *Spiroplasma* species [136–138], and are enriched for evolutionary changes in *s*Hy and *s*Mel (Fig. 2). In addition, we documented dynamic evolution and turnover of toxin loci, which are important for host fitness and symbiont compatibility (Figs 6 and S6). This genomic flexibility may contribute to *Spiroplasma *’s broader host range when compared with *Wolbachia *. However, elevated evolutionary rates also make deleterious changes more likely and, in the absence of strong selection, may result in faster loss of symbionts [139]. This may explain the generally low prevalence of *Spiroplasma* symbionts [15], which seems to increase only when carrying the symbiont is associated with a large fitness benefit [21]. In contrast, virtually identical *Wolbachia* strains (‘superspreaders’) are found in many different host species at very high frequencies [140] – demonstrating that stationary genomes may be evolutionary advantageous. In summary, the nature of *Spiroplasma* genomic evolution likely contributes to its peculiar evolutionary ecology.

From a practical perspective, *S. poulsonii* has many features of a desirable model for symbiont–host interactions: fast rates of evolution make it more likely that adaptation and spontaneous changes in phenotypes can be determined over short time scales, as has been observed previously [32, 35, 36]. However, fast evolutionary changes make experiments less predictable, and because stochastic effects become more pronounced, links of genomic changes with phenotypes may be obscured. Further, the generalisability of experimental results may be limited for extremely fast evolving symbionts. Our findings, therefore, also underline the importance of regular validation of laboratory symbiont strains through re-sequencing.

## Supplementary Data

Supplementary material 1

Supplementary material 2

Supplementary material 3

Supplementary material 4

Supplementary material 5
