# Supplementary material for: Rapid molecular evolution of Spiroplasma symbionts of Drosophila
Source: Microb Genom. 2021 Feb 16;7(2):000503. doi: 10.1099/mgen.0.000503 (PMC8208695; doi:10.1099/mgen.0.000503)
Supplement: Supplementary material 5 [file mgen-7-503-s005.pdf]

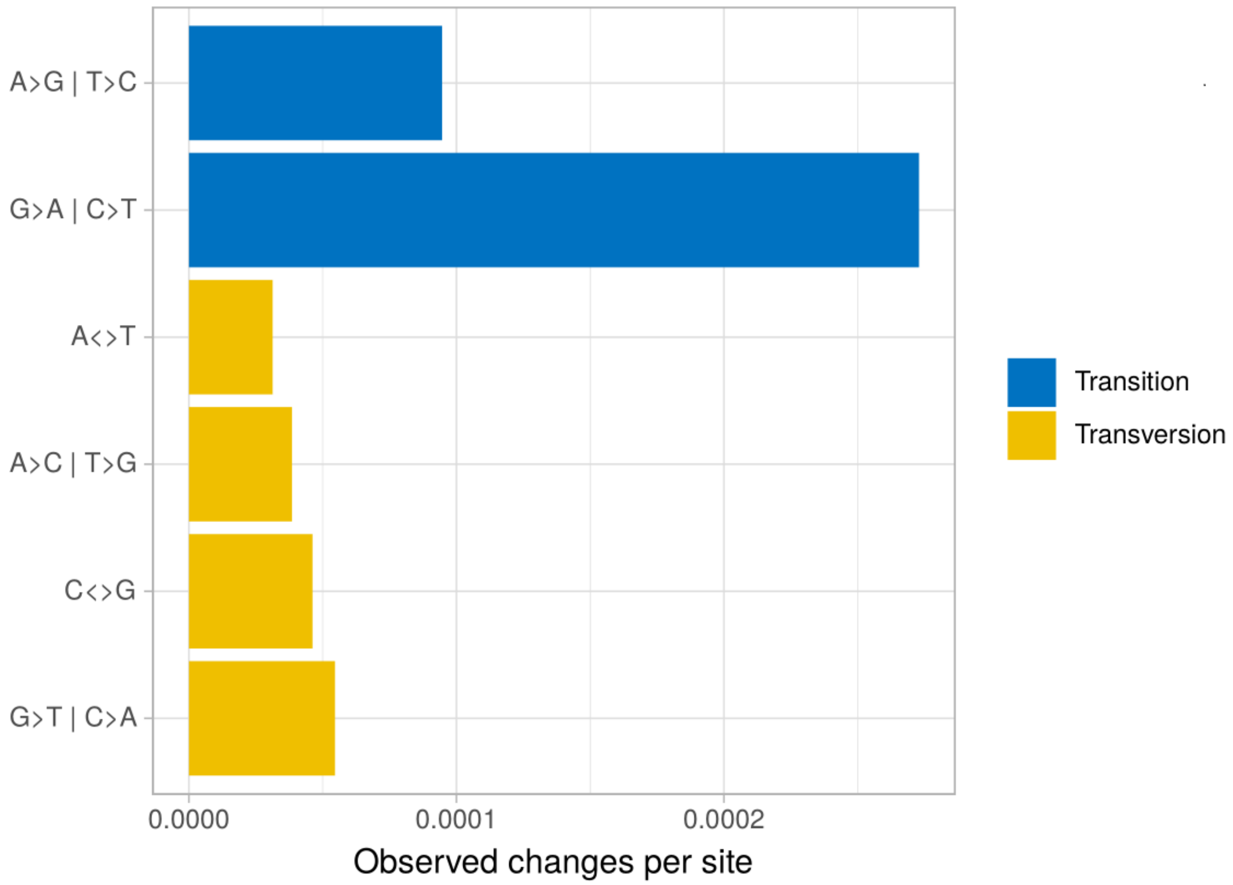

Figure S1. Mutational bias in *s*Hy evolution. Rates were calculated by counting the corresponding numbers of snps and dividing by the total number of AT or GC positions in the *s*Hy genome.

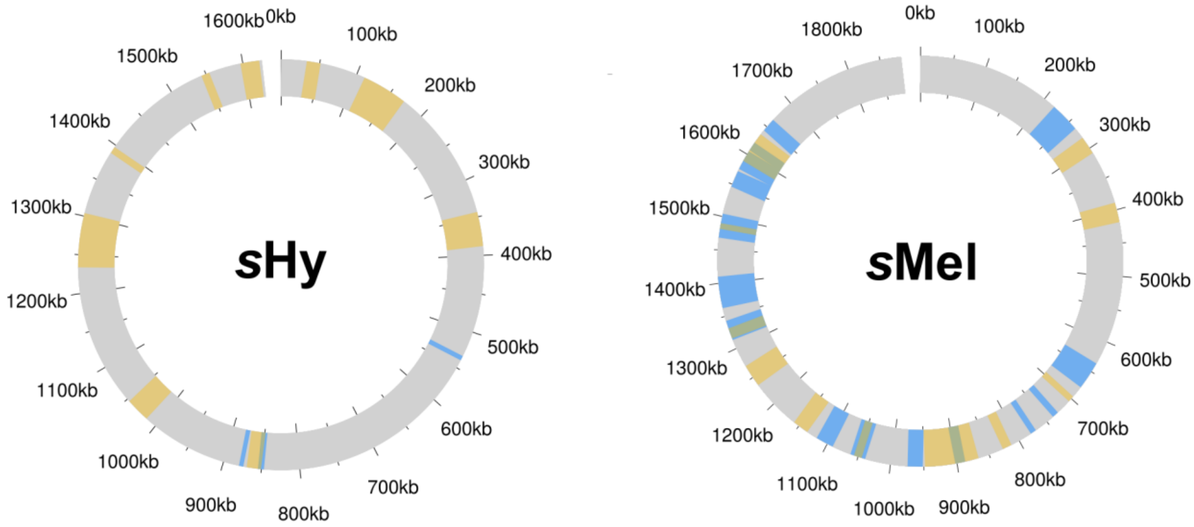

Figure S2. Predicted prophage regions in *s*Hy and *s*Mel by PHASTER (blue) and PhiSpy (yellow).

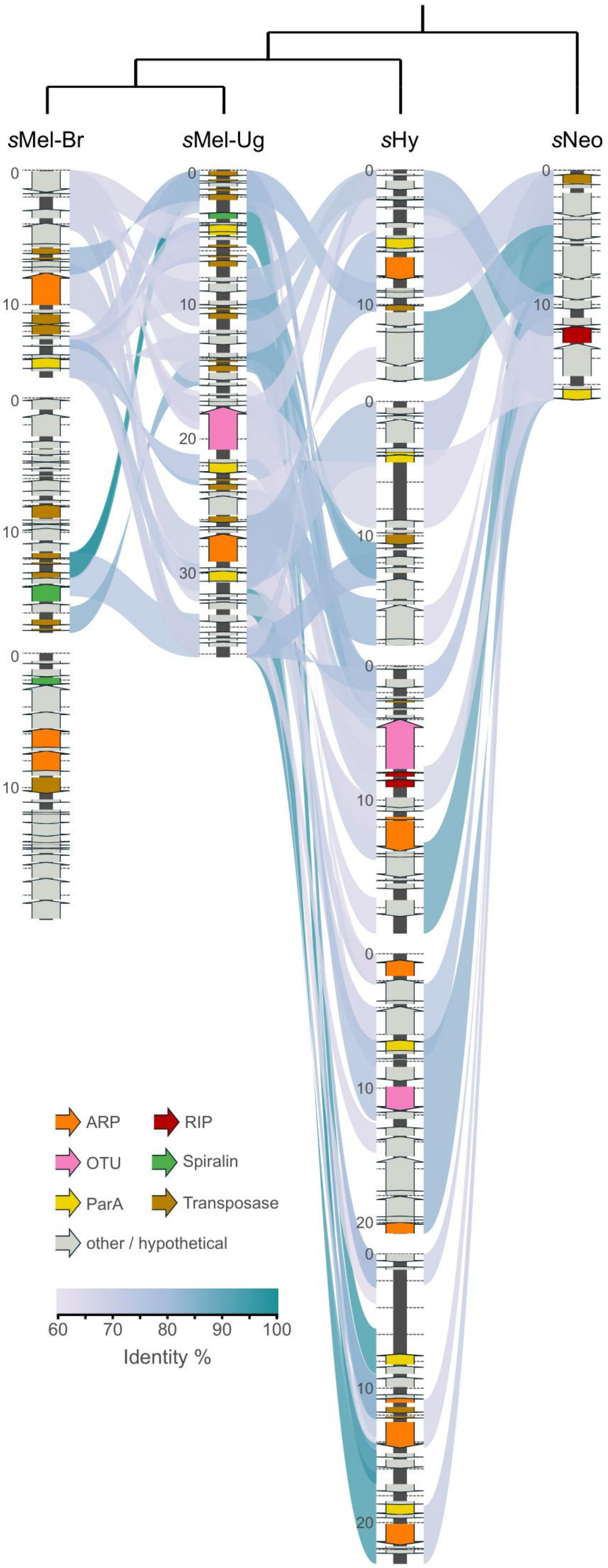
sMel-Br
sMel-Ug
sHy
sNeo
ARP
RIP
OTU
Spiralin
ParA
Transposase
other / hypothetical
60
70
80
90
100
Identity %

Figure S3. Plasmid synteny in *Spiroplasma poulsonii* strains. Numbers on ticks correspond to nucleotide position on plasmids in kb. Sequence similarities (>=60% and spanning >= 2kb) between plasmids are indicated by ribbons. Abbreviations: ARP - adhesion related protein, OTU - ovarian tumor domain containing protein (in *s*Mel: Spaid), ParA - plasmid partitioning protein like, RIP - ribosome inactivating domain containing protein, Spiralin - spiralin like protein. Figure was created with AliTV.

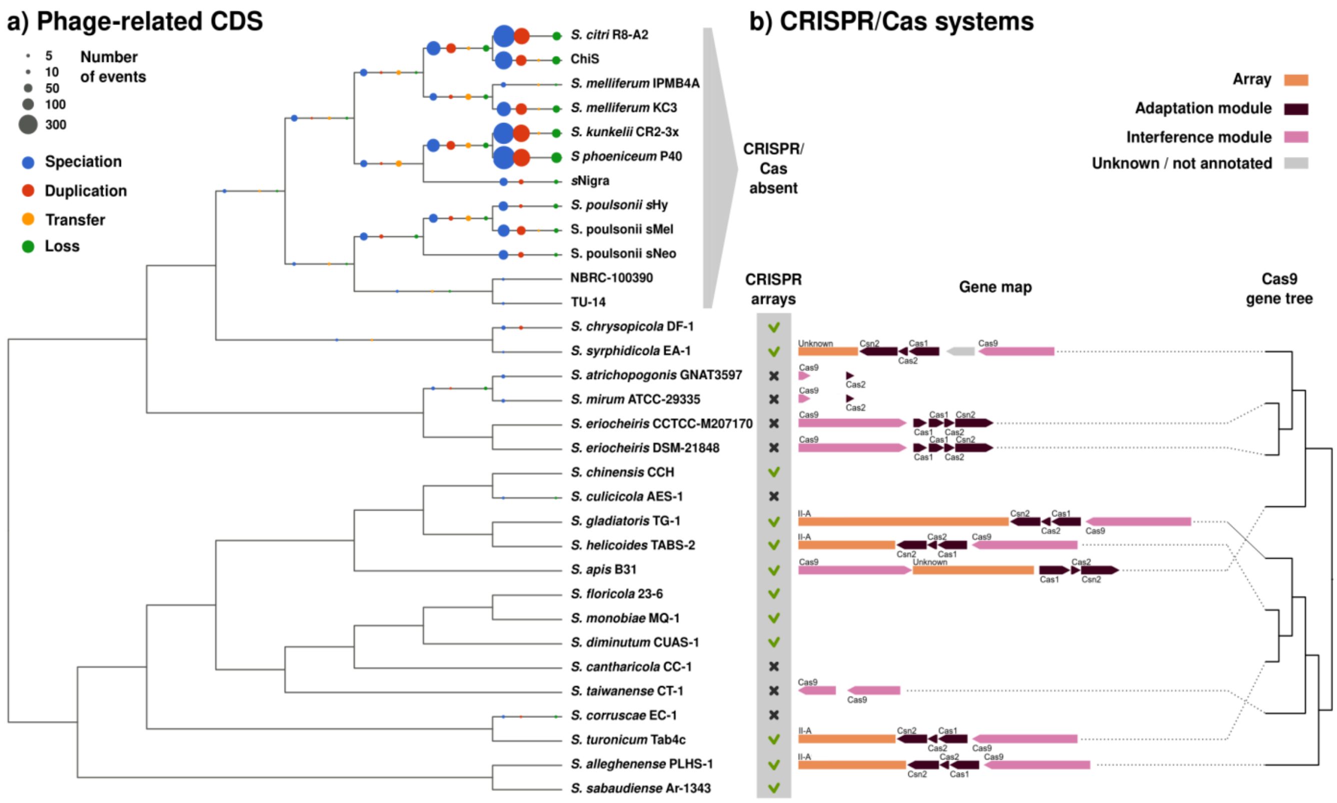

Figure S4. Prophage loci and CRISPR/Cas systems in *Spiroplasma*. **a)** Summary of prophage gene tree-species tree reconciliations. All events (gene speciations, duplications, transfers and

losses) were inferred using GeneRax and are mapped onto the *Spiroplasma* phylogeny based on single copy orthologs present in all strains. **b)** CRISPR/Cas systems and arrays as predicted by CCtyper and CRISPRidentify, respectively. Maximum likelihood phylogeny is based on aligned Cas9 protein sequences, and was reconstructed using IQ-TREE. Note that CRISPR/Cas is absent in all Citri and Poulsonii strains. Several strains have lost or reduced parts of the CRISPR/Cas system, which therefore are likely not functional.

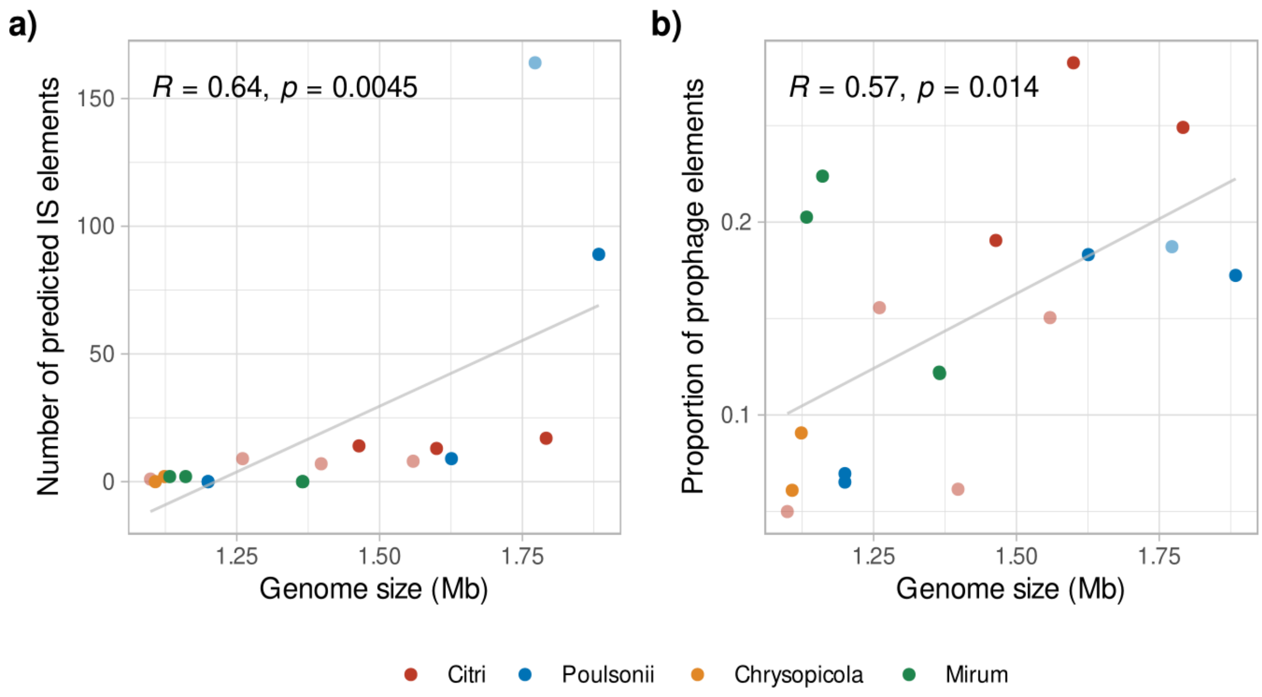

Figure S5. Correlations between genome size of investigated *Spiroplasma* strains and **a)** The number of IS elements as predicted by prokka; **b)** proportion of the genome encoding for prophages as predicted by PhiSpy. Note that draft assemblies are plotted using lighter colors, and correlations are similarly strong when excluding the draft assemblies: a) R = 0.77, p = 0.053; b) R = 0.57, p = 0.044. Abbreviations: R - Pearson's correlation coefficients; p - p-values.

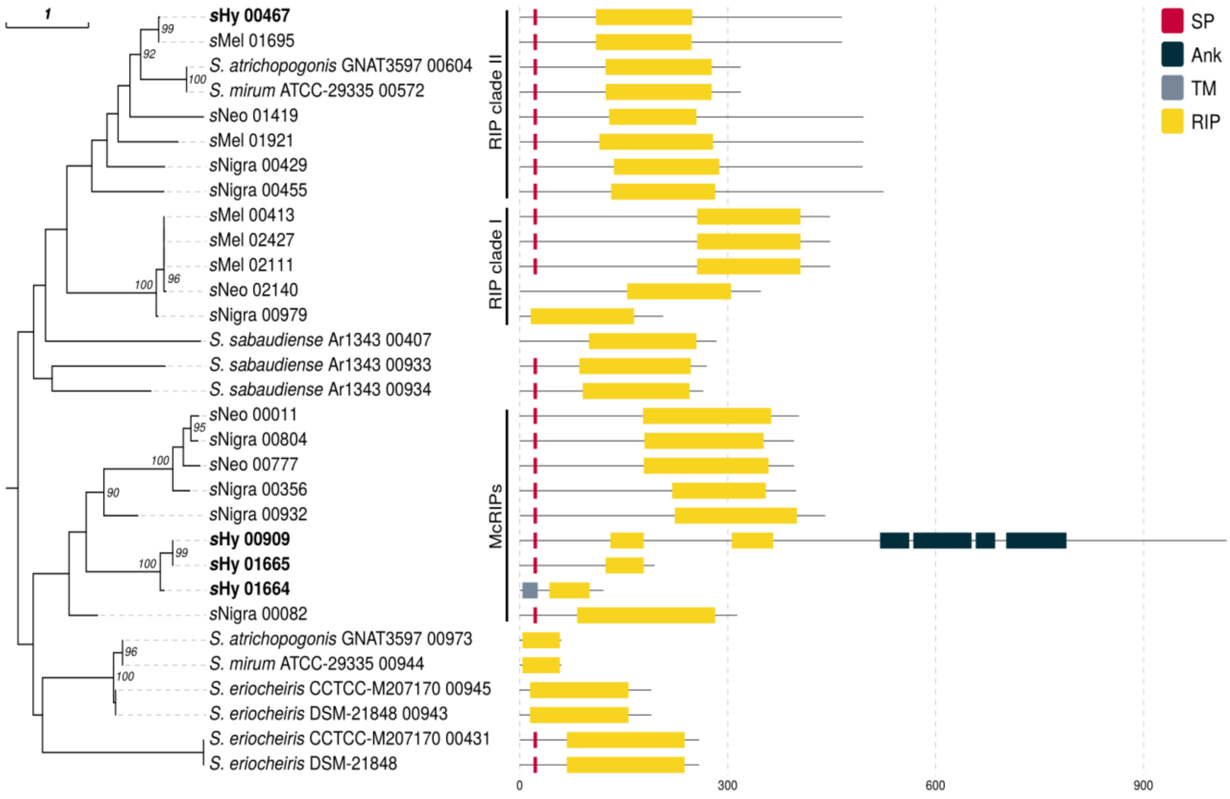

Figure S6. RIP loci in different *Spiroplasma* genomes. Maximum likelihood tree was calculated with IQ-TREE based on an alignment of RIP domains (288 amino acid positions), created using the hmmalign function of the HMMER software, and manually trimmed to exclude positions present in < 3 sequences. Domain prediction is based on PfamScan, SignalP, and TMHMM. Clades identified by Ballinger & Perlmann (2017) are indicated, and sequences from *s*Hy highlighted in bold font. UFB Bootstrap values ≥90 are shown on nodes. Abbreviations: SP - signal peptide, TM - transmembrane helix, Ank - ankyrin repeats, RIP - ribosome inactivating domain containing protein.
